# P-1697. Antibiotic prescribing patterns among outpatients with acute sinusitis in Michigan

**DOI:** 10.1093/ofid/ofae631.1863

**Published:** 2025-01-29

**Authors:** Walaiporn Wangchinda, Anne Haddad, Elisia Stier, Brenda M Brennan, Anurag Malani, Jason M Pogue

**Affiliations:** University of Michigan College of Pharmacy, Ann Arbor, Michigan; Michigan Department of Health and Human Services, Lansing, Michigan; Michigan Department of Health and Human Services, Lansing, Michigan; Michigan Department of Health and Human Services, Lansing, Michigan; Trinity Health Michigan, Ann Arbor, Michigan; University of Michigan, College of Pharmacy, Ann Arbor, MI

## Abstract

**Background:**

Acute sinusitis is a common presenting illness in the outpatient setting. While antibiotics are often prescribed, they are infrequently indicated in adults. Limited data are available assessing prescribing practices. This study aimed to describe statewide antibiotic prescribing for sinusitis among outpatients in Michigan to identify opportunities for antimicrobial stewardship (AMS) interventions for clinical and public health partners.

**Figure 1:**
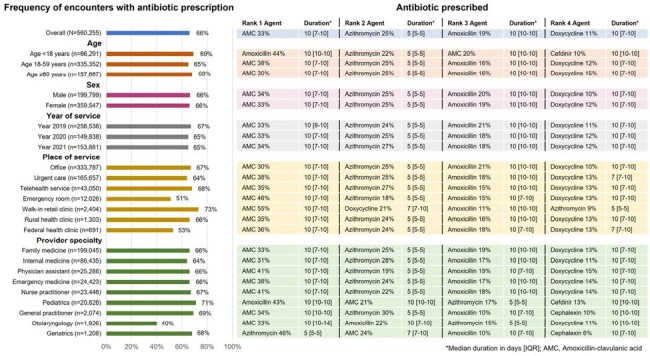
Outpatient antibiotic prescribing practices for sinusitis in Michigan, 2019-2021

**Methods:**

The Michigan Department of Health and Human Services received medical and pharmacy claims data from IQVIA from 2019 – 2021. Outpatients with medical claims data with a diagnosis code (J01) for acute sinusitis were included, while those with concomitant upper respiratory tract, urinary tract, or skin and soft tissue infectious diagnoses codes were excluded. Patients with a J01 diagnosis in their medical claims were linked to their corresponding antibiotic prescriptions from pharmacy claims based on patient identifier, prescription date, ordering provider, and antibiotic utilized. Prescribing patterns of antibiotics (frequency of prescription, agent selection, and duration of therapy) were analyzed for the overall cohort and stratified by place of service, provider specialty, year of service, patient zip code, age, and gender.

**Figure 2:**
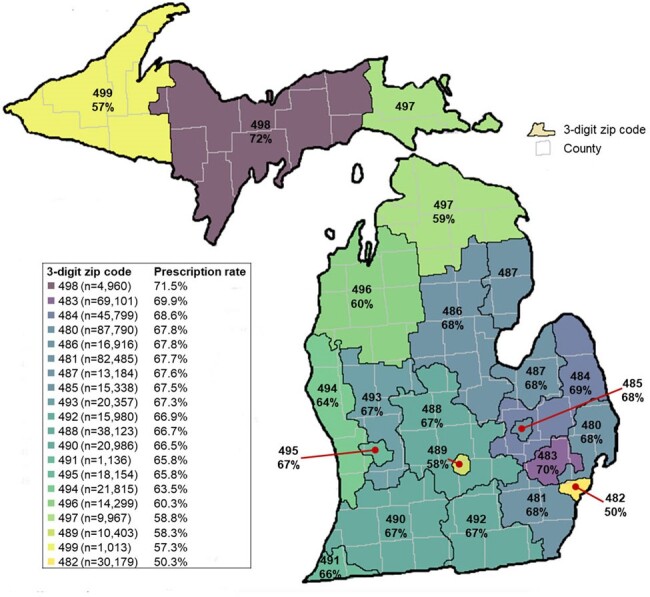
Frequency of acute sinusitis encounters with an antibiotic prescription by 3-digit zip code

**Results:**

From 2019 - 2021 there were 560,255 unique encounters from 424,684 patients for acute sinusitis in Michigan within the IQVIA medical claims database. Antibiotics were prescribed in 66% of these cases. Amoxicillin-clavulanic acid (33%), azithromycin (25%), and amoxicillin (20%) were the most prescribed antibiotics, with median durations of 10 days, 5 days, and 10 days, respectively. The prescription patterns among different groups are shown in **Figures 1** and **2**. Prescription rates, agent selection, and durations were remarkably consistent across calendar year, age group, sex, place of service, provider specialty, and patient zip code.

**Conclusion:**

The study revealed a concerning pattern of high antibiotic prescription rates, inappropriate antibiotic selection, and prolonged durations of therapy in the outpatient management of acute sinusitis in Michigan. These findings were consistent in all subgroups analyzed and emphasize the need to prioritize outpatient AMS practices for sinusitis.

**Disclosures:**

**jason M. Pogue, PharmD**, Entasis: Advisor/Consultant|GSK: Advisor/Consultant|Melinta: Advisor/Consultant|Melinta: Grant/Research Support|Merck: Advisor/Consultant|Merck: Grant/Research Support|Shionogi: Advisor/Consultant|Shionogi: Grant/Research Support|Venatorx: Advisor/Consultant

